# Round the Hospitals

**Published:** 1919-12-13

**Authors:** 


### ROUND THE HOSPITALS.

THE KING held an Investiture on December 3 in the Ballroom of Buckingham Palace, and personally conferred decorations on members of the Nursing Services as follows:—Bar to the Royal Red Cross: Miss Louisa Tulloh, Q.A.I.M.N.S. Royal Red Cross, First Class: Miss Elsie Schafer, Q.A.I.M.N.S.; Miss Marie Gilkes-Robinson, T.F.N.S. Royal Red Cross, Second Class: Miss Mary Tawney and Mrs. Nina Wynne, Q.A.I.M.N.S.; Miss Kezia Hawkins, Miss Helen Macdonald, Miss Susanna O'Halloran, Miss Daisy Rollo, Miss Emilie Rutledge, Miss Muriel Smith,

**ROUND THE HOSPITALS**—(*continued*).

Miss Mary Storey, Miss Constance Strange, Miss Amy Stuart, Miss Mary Tate, Miss Annie Thomas, Miss Ellen Walsh, Miss Mary Ward, Mrs. Ethel Watkins (who also received the Military Medal), and Miss Iris Withers, Q.A.I.M.N.S. (R.); Miss Janet Prentice and Miss Mira Walley, T.F.N.S.; Miss Marcia Conway-Gordon and Miss Anne O'Donoghue, Civil N.S. Military Medal: Miss Watkins and Miss Louisa Wilkinson, Q.A.I.M.N.S. (R.).

The King held a second Investiture last week in the Ballroom of Buckingham Palace on December 4, and personally decorated the following members of the Nursing Services:—The Most Excellent Order of the British Empire.—*Member*: Staff Nurse Barbara Banks, T.F.N.S. Royal Red Cross and Bar: Miss Ethel Collins, Q.A.I.M.N.S. Royal Red Cross, First Class: Miss Lilian Belcher, Q.A.I.M.N.S.; Miss Katie Payne-Hodge, Q.A.I.M.N.S. (R.). Royal Red Cross, Second Class: Sister Mary Hocking, Q.A.R.N.N.S.; Sister Nance McKay, Sister Maud Symonds, Miss Harriet Wells, and Miss Janet Williams, Q.A.R.N.N.S. (R.); Miss Sarah Smith, R. Naval Medical Service; Miss Ethel Clark, Miss Sylvia Corby, Miss Jessie Edgar, Miss Alice MacLean, Mrs. Mary Mair, Miss Mary McGeown, Miss Ethel Smith, Miss Mary Smith, and Miss Margaret Thomson, Q.A.I.M.N.S. (R.); Miss Mary Coupar, Miss Alice Sampson, and Miss Maud Smith, T.F.N.S.; Miss Lucy Brooks and Miss Fanny Pease, B.R.C.S. Military Medal: Miss Leila Thomson, T.F.N.S. All these ladies were subsequently received by Queen Alexandra at Marlborough House, when Miss A. B. Smith, R.R.C., Matron-in-Chief, was also a guest of her Majesty.

The Council of the Jubilee Institute, which met on Queen Alexandra's birthday, sent a message of congratulation to her Majesty, who sent a kind telegram in reply, with thanks "for kind birthday congratulations, which I much appreciate." A scheme for the reconstruction of the Council and Committee was approved. It will come into force next February, when the new Council will be appointed. Twenty-nine Associations have affiliated since the last meeting, and seventy-five nurses have been enrolled. We could wish it were seventy times seventy.

The recent announcement of Lord Knutsford that the London Hospital system of training is for the future to be based on a three years' course will cause great satisfaction to all who have the interests of nurses at heart. Ever since Lord Knutsford lent his support to the Registration Bill, introduced into the House of Lords last spring, it has been anticipated that a change of system would take place at the London Hospital, for it was inconceivable that "London" nurses should continue to remain outside the scope of registration. A general shortening of hours is also forecasted, though not a 48-hour week, which would entail an unprecedented enlargement of the staff and cost more money than could be allocated for this purpose. The change of system is heartily welcomed by the nurses in training, for, however satisfied probationers may feel with their surroundings, their teaching and their opportunities for advancement, it is not in human nature to be content with less than the very best, and this by common consent is not to be achieved short of three years' hospital work.

The Irish are quite determined that they shall not be left out of the Registration movement for nurses. At a recent meeting of the newly-formed Irish Public Health Council Dr. F. Coey Bigger, who was in the chair, drew attention to the Nurses' Registration Bill for England lately introduced into the House of Commons, and announced that he had been communicating with the Minister of Health for Ireland and the English Ministry of Health on the subject of a similar Bill for Ireland. He had also consulted the various nursing organisations in Ireland, and they had agreed generally to his recommendations. It is proposed that the Irish Bill shall be modelled on that now before Parliament, but with a much smaller council than that proposed for England. Irish nurses would be placed at a great disadvantage if the benefit of State registration were denied to them after this had been gained by their English *confrères*, and we trust nothing may retard the introduction of the proposed Bill.

The Council of the College of Nursing has awarded grants to fifteen members to enable them to obtain the Central Midwives Board Certificate. The Council has had to deal with no fewer than 116 applications for such grants, and it greatly regrets that so many have had to be refused. It is to be hoped that the lucky fifteen, when they have obtained the C.M.B. Certificate, will not fail to practise the art for which the College has thus enabled them to qualify themselves.

Some remarkable facts relating to the V.A.D. organisation of the British Red Cross Society were revealed at the great meeting of the branch presided over by the Duchess of Beaufort on November 15. The public of Bristol contributed nearly £100,000 to the branch during the war. The whole of the detraining of wounded and sick troops was carried out without a single mishap. From January 1918 to October 1919 the Society's ambulances conveyed 14,754 stretcher and 47,767 sitting cases, and dealt with 417 ambulance trains. The prisoners of war were systematically attended to, and in October 1918 there were over 1,000 men each receiving three food parcels a fortnight. It is a fine record, and the best of it is that the branch is eager to resume work and help to make England a fit place for heroes.

The exchange rate of payment has been the subject of much legitimate complaint by the military officer in India. But there is, it would appear, one class of very deserving individuals whose griev-

**ROUND THE HOSPITALS**—(*continued*).

ances on this point particularly call for prompt attention. We refer to the sisters and staff nurses of the Q.A.I.M.N.S., Q.A.I.M.N.S. Reserve, and Territorial Force Nursing Service, at present employed in Mesopotamia. The sisters receive pay varying from £50 to £65 per annum, and the staff nurses £40 to £45. To these sums have to be added staff pay for the duration of war engagements, and washing, field and special Mesopotamian allowances. Before the exchange value of the rupee began its sensational course upwards, the pay, with these allowances, worked out (at the maximum rates) for sisters to Rs.236-1 per mensem for a month of 31 days, or Rs.231-14 for a 30-day month; and for staff nurses to Rs.211 or Rs.206-14. The rise in the exchange value of the rupee to 1s. 8d. has meant a loss for these sisters of anything from Rs.441 to Rs.47 per mensem in the pay and allowances received, and this loss has been further augmented by the still further rise in the exchange to the 1s. 10d. level. If the same system of payment is adhered to exchange at 2s. will bring about a reduction of the sister's pay to less than Rs. 160, and of nurses to Rs. 140. In commenting on these points, a leading Indian journal says: the pay of the nursing service was small enough in all conscience in the beginning to make it certain that any reductions in the amount received would entail very great hardship. And whatever may be the technically correct reason for paying sisters and nurses their actual pay at the exchange rate, it cannot be right to pay their allowances at that rate. Because the rupee is worth more English money, it does not follow that food and washing and other expenses in Mesopotamia which have to be paid for in rupees will cost less. A further point in this connection is that the sisters do not receive any war bonus to correspond to that granted weekly to all British troops in the armies of occupation. The facts above given surely speak for themselves.

---

"My visit to Norway," which started on the first day of the railway strike, was the subject of an address of vivid interest which Miss Sparshott, C.B.E., R.R.C., delivered to the members of the East Lancs Centre (Manchester Branch) of the College of Nursing on November 25. The visit was arranged by the Norwegian Red Cross, who invited 100 British nurses to stay for one month in hotels or with British-speaking families outside Christiania, the invitation including a free passage from Newcastle to Bergen, and free travelling in Norway. Miss Sparshott's account of how she and her companion were stranded the first night at Normanton and then had to proceed by taxi to York and thence by another taxi to Newcastle at a cost of £15 10s., her determination to be prepared for any emergency, her consequent purchase of a "trousseau" of mufti, though she found afterwards uniform only was to be worn, and a hurried visit to London for passports, showed the enthusiasm which characterised her and Miss Woodhouse (the other representative of the Unit) on this memorable visit. Miss Sparshott illustrated her lecture by lantern-slides, and gave a terse but informative account of Norwegian customs, manners, scenery, social life, and education. Miss Sparshott, with three other principal matrons, were present at the birthday party of General Daacs, the Chief of all medical matters in the Norwegian Army, and were invited to the Palace by Queen Maud, where they also saw the King and Prince Olaf. The Norwegian people showed themselves most hospitable and friendly to their guests, and the visit included many social functions, both to Norwegian and English families, all of which Miss Sparshott described in her most spirited and interesting address.

---

The Bristol General Hospital is fortunate in having the support of a specially well organised and enthusiastic Ladies' Needlework Guild, and a very good year's work was evidenced when the annual meeting of the guild took place on November 6. The articles made numbered 2,386, and in addition 71 articles had been purchased for the use of patients. Besides this, sums amounting to £192 had been collected. The hospital was thrown open to visitors for the occasion and Miss Densham, the matron, provided tea in the nurses' lounge.

---

The Ladies' Needlework Guild of the Royal United Hospital, Bath, has a splendid achievement to its credit. Thanks to the energy of Mrs. Handley, the founder, and of the many associates and helpers, the Guild has freed the funds of the hospital from all expense in connection with the linen for the whole of the institution for twenty-five years. During the past year it received linen to the value of £150 and £142 in cash, and closed with a balance in the bank of £215.

---

The seventeen members of the Scottish Women's Hospital who died on active service in France and Serbia were commemorated on St. Andrew's Day by the placing on the cenotaph of a wreath in their memory from the ninety-eight members of the Royaumont Association of Old Comrades. These ninety-eight members held their first reunion and annual dinner on the same day, and, in memory of the founder of the Scottish Women's Hospital, Dr. Elsie Inglis, a vacant chair, wreathed with laurels, was placed at the table. During the Scottish Women's Hospital's Week, beginning on December 15, it is hoped to collect funds for the national memorial to Dr. Inglis, which will include the permanent financing of her maternity and child-welfare work in Edinburgh and the foundation in Serbia of a hospital with a training-school for nurses attached. We note that the offices of the National Memorial Fund are at 110 Victoria Street, S.W. 1.

---

Another field for work is opened up by the Education Act in the section giving powers for the formation of nursery schools. Local education authorities are here given full scope for

**ROUND THE HOSPITALS**—(*continued*).

experimental work. Now is their opportunity before they become bound up in official red tape and tied to stereotyped syllabuses. The work should not be carried out except in close co-operation with the adjacent infant welfare centres, and one cannot help feeling that an important service to the country would be rendered if the principle of cleanliness were thoroughly inculcated into the parents and even the elder children. It is extraordinary how readily even small children will come to be of assistance in this cause. At a school outside the London area a teacher recently offered a small reward to each child who should come back to school after the holidays quite clean. At the beginning of the term every child passed the school nurse. If a child really wishes anything the parent can usually be induced to supply it, and the main thing is to rouse the desire to be clean, for this is the first step to the habit of cleanliness.

---

THE Committee of the West Kent General Hospital, Maidstone, is erecting a new sectional Army hut which is about to be opened. It is equipped as a massage, remedial exercise, and electrical department for the treatment of the hospital's patients and the large number of Army pensioners. The department will be staffed with a head masseuse and four or five assistants. Miss E. M. Greenall, B.Sc. (Lond.), has been appointed head masseuse at a salary of £200 per annum, and the full-time assistants will be paid £150 per annum. Miss Greenall has the certificates of the Incorporated Society of Trained Masseuses in Massage, Swedish Remedial Exercises, Medical Electricity and Radiography. Through the generosity of Canon and Mrs. Levett an outdoor shelter containing five cots has also been erected adjoining the Children's Wards. It will undoubtedly prove of great value in the treatment of the child patients.

---

A VERY pleasant ceremony was carried out at the annual meeting of the Ipswich Nurses' Home on November 21, under the chairmanship of Major Mellor, when a purse containing forty-five guineas was presented to Miss Pye, who has been connected with the Home for forty-five years. It was started under Miss Pye's matronship when smallpox was rife in the town with ninety-five cases in one street. From that time on the Nurses' Home has done fine work in the town, and the traditions of personal devotion have been carried on unbroken. Sister Blyth, who has been acting matron for the last five years in the absence of Miss Newton on war duty, has now been appointed matron, with Sister Beadle as her assistant. The nurses undertake district, school and private nursing, and the isolation block is now used as a maternity home, where 50 cases were treated in the past year. There is also a surgical nursing home. It is satisfactory to learn that salaries have been raised on a sliding scale depending on length of service, in lieu of a bonus system.

OWING to the serious impasse created at the Launceston General Hospital, Hobart Town, by the disagreement between the Government and the British Medical Association, the medical staff is reduced to one, and the matron, seven sisters, and seventeen nurses have taken the unusual course of writing to protest to the Hospital Board. The sisters and nurses ask: "Does it seem fair to the nurses who have nearly finished their training, that the surgical work which they have looked forward to during their training and of which the hospital has always been justly proud, is not to be part of their course?" The matron fully supports the feeling of the sisters and senior nurses, and the chairman on his part endorses to the full the petition and the matron's letter which were forwarded to the Premier.

---

AT a meeting of the Kingston Board of Guardians it was decided to ask the Ministry of Health to add ten years to the matron's services for superannuation purposes, thus making her retiring allowance £93, instead of £60. Miss Smith, who has been in the service of the Board for eighteen years, has been in indifferent health for some time, and is leaving the infirmary as soon as her health permits. Miss Smith's efficiency and capacity, her indefatigable energy and whole-hearted devotion, were cordially recognised at the meeting in question by the Vice-Chairman, Canon Challacombe, who said he could not speak in too high praise of these qualities.

---

THE Bradford Chamber of Commerce Council has decided, after an earnest appeal from their President, Mr. H. Sutcliffe Smith, to start a fund in Bradford for the French Red Cross Society. The Chamber has already raised £38,429 for the aid of the devastated area, and it is proposed to make a further thank-offering from the whole textile trade for its immunity from the heart-breaking tragedy which has overtaken their French brethren. Bradford citizens visiting Vitry, where the work is centred, are full of admiration at what British women are doing there, and in particular British nurses. There is an excellent dispensary at the depôt, under the charge of a nursing sister, with other nurses doing valuable work, as there is no doctor for many miles. Eight of these depôts have been established hitherto, but another thirty are urgently required.

---

STOBHILL Hospital, Springburn Park, Glasgow, has now been returned by the military authorities to the Glasgow Parish Council, and will soon be ready to receive patients. The training of nurses has been perforce suspended during the war, but under present conditions all probationer nurses will receive a training of four years, and applications can now be made to the Matron, Miss Merchant, whose appointment we announce in another column. The salaries at present paid by the Parish Council to nurses in training are: First year £24, second year £33 10s., third year £43, fourth year £62, with board, lodging, and uniform.

